# Relationship of Routine Inadequate Sleep Duration and Periodontitis in a Nationally Representative Sample

**DOI:** 10.1155/2016/9158195

**Published:** 2016-01-20

**Authors:** R. Constance Wiener

**Affiliations:** ^1^Department of Dental Practice and Rural Health, School of Dentistry, West Virginia University, G110B HSC North, P.O. Box 9448, Morgantown, WV 26506, USA; ^2^Department of Epidemiology, School of Public Health, West Virginia University, G110B HSC North, P.O. Box 9448, Morgantown, WV 26506, USA

## Abstract

*Purpose*. Previous research has indicated the public health impact of inadequate sleep duration on health, potentially through an immune-inflammation mechanism. This mechanism also has a role in periodontitis. The purpose of this study is to determine if there is an association of routine inadequate sleep and periodontitis.* Methods*. Data from merged National Health and Nutrition Examination Survey years 2009-10 and 2011-12 were the data source for the study. The key outcome was periodontitis (yes, no), and the key variable of interest was usual sleep on weekday or workday nights. Chi square and logistic regression procedures were conducted. The study included 3,740 participants who were of ages 30 years and above.* Results*. There were 52.7% of participants who had periodontitis. There were 35.7% who usually slept less than 7 hours on weekday or workday nights. In adjusted logistic regression the odds ratio for periodontal disease for participants who slept less than 7 hours on weekday or workday night was 1.00 [95% confidence interval: 0.83, 1.21; *p* = .9812].* Conclusions*. The relationship of periodontitis and inadequate sleep duration in a nationally representative study of participants who were of ages 30 years and above failed to reach statistical significance in adjusted logistic regression analyses.

## 1. Introduction

Periodontitis involves inflammation of gingival tissue, clinical attachment loss of the periodontal ligament, and loss of alveolar bone support. Periodontitis is a public health concern since it is a major factor in tooth loss [1] and has associations with many systemic diseases which also involve inflammation. In periodontitis, the host response to microbial pathogens leads to inflammation. The inflammation has been shown to result from (1) plaque and local factors overwhelming the host responses; (2) an inadequate host response in individuals with limited plaque and local factors but are compromised/susceptible individuals [2], (including smokers, people who have diabetes, immunosuppressed individuals, older adults, individuals with genetic redisposition for an inadequate host response, and individuals with excessive stress [3, 4]), and (3) an overwhelming host response [5]. An appropriate host response is required for defense of the tissue; the host defense must not be inadequate or overwhelming [5]. Identifying and eliminating risk factors which influence inflammation may provide means by which to limit periodontal tissue destruction.

It is known that sleep is important to health and well-being. Inadequate sleep has effects on learning, memory processing, the repair of cell damage, brain development, neurobehavioral performance, hormonal regulation, risk of depression, increased cortisol, and ghrelin, impaired glucose metabolism, and increased inflammatory and proinflammatory markers among many other influences [6]. It is also known that there has been a worldwide decrease in the average number of hours that people sleep since the mid-1970s. Currently, the average number of hours that a person sleeps is less than seven hours a night [6]. Both adults and children are sleeping fewer hours than adults and children of the previous generation [6].

Since inflammation is characteristic of both periodontitis and inadequate sleep, sleep may have a role in periodontal disease. The purpose of this study is to determine if there is an association of sleeping less than 7 hours a night and periodontitis in individuals of ages 30 years and above.

## 2. Methods

The present study received West Virginia University Institutional Review Board acknowledgement (protocol number 1510880211).

### 2.1. Data Source

The data sources for the present study were National Health and Nutrition Examination Surveys (NHANES) 2009 to 2010 and NHANES 2011 to 2012. The Centers for Disease Control and Prevention researchers for the NHANES used stratified, multistage probability sampling designs for the surveys. The NHANES participants were civilians who were noninstitutionalized and lived in the US and Washington, DC. The researchers oversampled smaller subgroups to increase estimate accuracy. Data for the full mouth periodontal examination were collected in a mobile examination center by calibrated examination using Hu Friedy PCP-2 (Hu Friedy, Chicago, IL) periodontal probes with markings of 2–4 mm, 6 mm, and 10–12 mm parallel to the tooth's long axis [7, 8]. The examiners were licensed dental hygienists in 2009-10 [8] and licensed dentists in 2011-12 [9]. Participants were of ages 30 years and above. The participants also responded to interview questions involving demographic information and questions regarding health and nutrition. Details of the NHANES study are available at the NHANES websites.

### 2.2. Study Population

The data used for the present study were of participants who were of ages 30 years and above who had complete periodontal and sleep data in the NHANES 2009 to 2012 data sets.

### 2.3. Key Outcome Variable

The key outcome variable was periodontitis (yes, no). The presence of any periodontitis was defined as at least 2 interproximal sites with an attachment loss of at least 3 mm and at least 2 interproximal sites with probing depths of at least 4 mm which are not on the same tooth or at least one site with a probing depth of at least 5 mm, suggested by the Centers for Disease Control and Prevention and the American Academy of Periodontology for use in research/surveillance of periodontitis for at least mild periodontitis [10].

### 2.4. Key Variable of Interest

The key variable of interest was routine adequate sleep. Participants were asked about the number of hours usually slept on a weekday or workday night. The self-reported responses were dichotomized at 7 hours based upon previous research using 7 hours [11] and for the premise of prevalent, routine inadequate sleep.

### 2.5. Other Variables

Participants were also asked during an interview to report age, sex, race, education level, and smoking status among other questions. The variables were categorized as follows: age (45 years to less than 55 years, 55 years to 69 years); sex (male, female); race/ethnicity (non-Hispanic White, non-Hispanic Black, Mexican-American, etc.); education level (high school graduate or less, some college/technical school or above); and smoking status (current smokers, former smokers, and never smokers). Body mass index was categorized as less than 25, 25 to less than 30, and 30 and above which corresponded to normal weight, overweight, and obesity.

### 2.6. Statistical Analyses

SAS^*®*^ version 9.3 (SAS Institute, Inc., Cary, NC) was used to determine sample descriptions, bivariate associations of the variables of interest with periodontitis, and logistic regression of inadequate sleep on periodontitis in both unadjusted and adjusted analyses. The analyses accounted for stratification, eligibility, and sample weights.

## 3. Results

The sample size for the present study was 3,740 participants. There were 1,873 participants who were women, a weighted row percentage (wt%) of 50.8. There were 1,790 participants (71.7 wt%) who were non-Hispanic White. There were 1,454 participants (32.8 wt%) who were of ages 55 years and above. The majority of participants (60.9 wt%; *n* = 1,890) had some education beyond having graduated from high school. There were 728 participants (17.0 wt%) who were current smokers.

There were 1,646 participants (52.7 wt%) who had periodontitis. There were 1,484 participants (35.7 wt%) who usually slept less than 7 hours at night on weekdays or workdays. Details of the sample are presented in Table 1.

The results of the bivariate analysis of periodontitis and the sleep variable as well as the analyses of periodontal disease and other variables are presented in Table 2. There was a significant Rao Scott Chi square association of periodontitis and usually sleeping less than 7 hours at night on weekdays or workdays. Other significant relationships with periodontitis were with diabetes; older age; male sex; being non-Hispanic Black, Mexican American, or other (as compared with being non-Hispanic White); lacking insurance coverage; being a current or former smoker (as compared with being a never smoker); having a lower federal poverty level; and having an education of high school graduation or less as compared with some college, technical school, or more.

The results of the logistic regressions of the sleep variable on periodontitis are presented in Table 3. In unadjusted analysis, usually sleeping less than 7 hours at night on weekdays or workdays was associated with periodontitis. The odds ratio was 1.34 [95% confidence interval: 1.18, 1.52; *p* < .0001]. The association was attenuated and failed to reach significance in adjusted analysis. The adjusted odds ratio was 1.00 [95% confidence interval: 0.83, 1.21; *p* = .9812]. The other variables remained significant in the adjusted analysis, except for diabetes.

## 4. Discussion

The purpose of this study was to determine if there was an association of sleeping less than 7 hours a night and periodontitis in individuals who were of ages 30 years and above. There was a significant positive adjusted association of inadequate sleep, defined as usually sleeping less than 7 hours a night on periodontitis in unadjusted analysis; however, the adjusted odds ratio failed to be significant. The adjusted logistic regression analysis included adjustments for age, sex, race, education, diabetes, insurance coverage, number of missing teeth, federal poverty level, and smoking.

### 4.1. Other Studies

There are few studies with which compare the results of this current study; however, studies of the quality of sleep (rather than sleep quantity) and severe periodontal disease (rather than no periodontitis versus any periodontitis) were found to be associated with periodontal disease:

(i) A study of 60 participants, ages 25 to 50 years living in Punjab, in which the mean Pittsburgh Sleep Quality Index results were highest for individuals with chronic periodontal disease [4].
(ii) A case-control study of individuals with newly diagnosed nonapnea sleep disorders (ages 18 to 95 years living in Taiwan), the incidence rate ratio of severe periodontal disease was 39% higher in the individuals with newly diagnosed nonapnea sleep disorders as compared with the control group, and the adjusted hazard ratio was 1.36 (95% CI: 1.04, 1.24; *p* < .001) [12].
(iii) A study of sleep disordered breathing in the Hispanic Community Health Study/Study of Latinos (ages 18–74 years, *n* = 12,469) in which researchers reported a positive relationship of severe periodontitis and increasing sleep disordered breathing (*p* < .001) in an adjusted analysis [13].

### 4.2. Biological Plausibility

Sleep interferes with the immune system and the inflammatory response [4] and in a study of 30 adults who were sleep deprived (sleep limited to 3 a.m. to 7 a.m.), researchers found monocyte production of interleukin-6 and tumor necrosis factor alpha to be significantly increased [14]; however, in a study of 19 participants who underwent 40 hours of total sleep deprivation, although there was a significant increase in interleukin-1beta and interleukin-1ra, there was a significant decrease in c-reactive protein and interleukin-6 [15]. There is a need to further study the influence of routine sleep patterns upon the biological markers.

### 4.3. Limitations and Strengths

A cross-sectional design does not include temporality or causation. There may be potential confounders that have not been considered in the adjusted analysis. Nevertheless, this study used 2009–2012 data from a large, nationally representative survey in which the periodontal examinations were performed by calibrated, licensed dentists and dental hygienists and questionnaires were presented by trained examiners.

## 5. Conclusion

The relationship of periodontitis and routine inadequate sleep duration in a nationally representative study of participants who were of ages 30 years and above failed to reach statistical significance in adjusted analyses.

## Figures and Tables

**Table 1:** Sample characteristics: NHANES Health and Nutrition Examination Surveys 2009–2012.

Total	Frequency 3,740	Weighted percent 100
Periodontitis		
No	1,646	52.7
Yes	2,094	47.3
Adequate sleep		
<7 hours	1,484	35.7
7 or more hours	2,256	64.3
Age groups		
30 to less than 45 years	1,341	38.5
45 to less than 55 years	945	28.7
55 years and above	1,454	32.8
Sex		
Female	1,873	50.8
Male	1,867	49.2
Race/ethnicity		
Non-Hispanic White	1,790	69.4
Non-Hispanic Black	671	11.0
Mexican American	676	8.0
Other	328	50.7
Education		
HS graduate or less	1,841	39.1
Some coll/tech/or more	1,890	60.9
Smoking		
Current	728	17.0
Former	952	26.0
Never	2,060	57.0
Number of missing teeth		
0–5	1,834	55.9
6–9	903	24.7
10–20	662	12.9
More than 20	341	6.4
Federal Poverty Level Index		
0 to less than 1.25	939	16.0
1.25 to less than 2.00	538	12.6
2.00 to less than 4.00	906	29.9
4.00 and above	962	41.5
Insurance coverage		
Yes	2,835	81.7
No	905	18.3
Diabetes		
Yes	559	11.3
No	3,181	88.7

Abbreviations: NHANES-National Health and Nutrition Examination Surveys; HS = high school; coll = college; tech = technical school.

**Table 2:** Bivariate relationships with periodontitis, NHANES 2009–2012.

Periodontitis	No		Yes		*p* value
	*N*	wt row%	*N*	wt row%	
Adequate sleep					<.0001
<7 hours	609	48.6	875	51.4	
7 or more hours	1037	55.0	1219	45.0	
Age groups					<.0001
30 to less than 45 years	837	69.6	504	30.4	
45 to less than 55 years	394	49.9	551	50.1	
55 years and above	415	35.4	1039	64.6	
Sex					<.0001
Female	1015	61.6	858	38.4	
Male	631	43.6	1236	56.4	
Race/ethnicity					<.0001
Non-Hispanic White	914	57.3	876	42.3	
Non-Hispanic Black	245	41.2	426	58.8	
Mexican American	212	33.4	464	66.6	
Other	275	49.3	328	50.7	
Education					<.0001
HS graduate/less	610	40.4	1231	59.6	
Some coll/tech/more	1033	60.7	857	39.3	
Smoking					<.0001
Current	221	35.7	507	64.3	
Former	372	47.5	580	52.5	
Never	1053	60.2	1007	39.8	
Number of missing teeth					<.0001
0–5	1,023	63.3	811	36.7	
6–9	388	50.3	515	49.7	
10–20	152	25.3	510	74.7	
More than 20	83	25.1	258	74.9	
Federal Poverty Level Index					<.0001
0 to less than 1.25	312	35.7	627	64.3	
1.25 to less than 2.00	228	44.3	355	55.7	
2.00 to less than 4.00	393	49.8	513	50.2	
4.00 and above	573	64.6	389	14.7	
Insurance coverage					<.0001
Yes	1349	56.2	1486	43.8	
No	297	37.0	608	63.0	
Diabetes					<.0001
Yes	151	33.4	408	66.6	
No	1495	55.2	1686	44.8	

Abbreviations: NHANES-National Health and Nutrition Examination Surveys; *N* = number of participants; wt% = weighted percentage; HS = high school; coll = college; tech = technical school.

**Table 3:** Logistic regressions of adequate sleep on periodontitis, NHANES 2009–2012.

	Unadjusted odds ratio	*p* value	Adjusted odds ratio	*p* value
	[95% confidence interval]		[95% confidence interval]	
Adequate sleep		<.0001		.9812
7 hours or more	Reference		Reference	
Less than 7 hours	1.34 [1.18, 1.52]		1.00 [0.83, 1.21]	
Age group				<.0001
30 to less than 45 years			Reference	
45 to less than 55 years			2.77 [2.04, 3.76]	
55 years and above			5.49 [3.77, 8.01]	
Sex				<.0001
Female			Reference	
Male			2.60 [2.17, 3.12]	
Race/ethnicity				
Non-Hispanic white			Reference	
Non-Hispanic black			1.88 [1.20, 2.96]	.0060
Hispanic			2.94 [2.18, 3.97]	<.0001
Other			1.65 [1.13, 2.41]	.0092
Education				<.0001
HS graduate/less			1.34 [1.09, 1.65]	
Some coll/tech/more			Reference	
Smoking				
Current			2.74 [1.98, 3.80]	<.0001
Former			1.43 [1.15, 1.79]	.0016
Never			Reference	
Number of missing teeth				
0–5			Reference	
6–9			1.44 [1.14, 1.82]	.0024
10–20			2.64 [2.02, 3.44]	<.0001
More than 20			2.18 [1.02, 4.65]	.0445
Federal Poverty Level Index				
0 to less than 1.25			1.82 [1.23, 2.71]	.0028
1.25 to less than 2.00			1.62 [1.15, 2.26]	.0053
2.00 to less than 4.00			1.60 [1.07, 2.39]	.0213
4.00 and above			Reference	
Insurance coverage				<.0001
Yes			Reference	
No			1.73 [1.43, 2.10]	
Diabetes				.1567
Yes			1.41 [0.88, 2.26]	
No			reference	

Wald test *p*-value was <.0001 for unadjusted and adjusted models.

Abbreviations: NHANES-National Health and Nutrition Examination Surveys; HS = high school; coll = college; tech = technical school.
